# Reimbursement for injury-induced medical expenses in Chinese social medical insurance schemes: A systematic analysis of legislative documents

**DOI:** 10.1371/journal.pone.0194381

**Published:** 2018-03-15

**Authors:** Yuyan Gao, Li Li, David C. Schwebel, Peishan Ning, Peixia Cheng, Guoqing Hu

**Affiliations:** 1 Department of Epidemiology and Biostatistics, Xiangya School of Public Health, Central South University, Changsha, China; 2 Department of Psychology, University of Alabama at Birmingham, Birmingham, Alabama, United States of America; TNO, NETHERLANDS

## Abstract

Social medical insurance schemes are crucial for realizing universal health coverage and health equity. The aim of this study was to investigate whether and how reimbursement for injury-induced medical expenses is addressed in Chinese legislative documents relevant to social medical insurance. We retrieved legislative documents from the China National Knowledge Infrastructure and the Lawyee databases. Four types of social medical insurance schemes were included: urban employee basic medical insurance, urban resident basic medical insurance, new rural cooperative medical system, and urban and rural resident medical insurance. Text analyses were conducted on all identified legislative documents. As a result, one national law and 1,037 local legislative documents were identified. 1,012 of the 1,038 documents provided for reimbursement. Of the 1,012 documents, 828 (82%) provided reimbursement only for injuries without a legally responsible person/party or not caused by self-harm, alcohol use, drug use, or other law violations, and 162 (16%) did not include any details concerning implementation. Furthermore, 760 (92%) of the 828 did not provide an exception clause applying to injuries when a responsible person/party could not be contacted or for situations when the injured person cannot obtain reimbursement from the responsible person/party. Thus, most Chinese legislative documents related to social medical insurance do not provide reimbursement for medical expenses from injuries having a legally responsible person/party or those caused by illegal behaviors. We argue that all injury-induced medical expenses should be covered by legislative documents related to social medical insurance in China, no matter what the cause of the injury. Further research is needed to explore the acceptability and feasibility of such policy changes.

## Introduction

Social medical insurance programs have long been used by state governments to protect finances of individuals and households affected by illness and injury and to realize universal health coverage for all citizens.[1] However, only social medical insurance schemes with appropriate reimbursement policies are able to adjust for income gaps, sharing financial risks across all citizens and increasing social equity.[2, 3]

Injuries are a significant global health challenge. In 2015, injuries caused 64.1 deaths per 100,000 population and accounted for 8.5% of all deaths in the world.[4] According to reimbursement principles of social medical insurance, patients affected by injuries should have the same right to receive financial subsidy as patients affected by infectious or non-communicable diseases. Unfortunately, this may not occur in all countries, primarily because injury control is seriously neglected in many countries including China.[5, 6]

China—which contains close to 1/5 of the world’s population—recently realized coverage of social medical insurance to over 95% of its residents, including to rural areas[7] The ongoing social medical insurance system in China is based on four kinds of schemes. The first is urban employee basic medical insurance (UEBMI), which was launched in 1998 by the State Council of China to relieve employees’ economic burden from diseases; it continued the earliest free medical care system for urban employees.[8] Because the traditional rural cooperative medical system collapsed in late 1980s, China initiated a second scheme, the new rural cooperative medical system (NRCMS), in 2003 as a voluntary insurance program to protect the vast uninsured rural population.[8] Subsequently, a third national social medical scheme, urban residents’ basic medical insurance (URBMI), was developed to cover students, children, and other unemployed urban residents who were not included in the UEBMI and NRCMS schemes.[8] As the implementation of the three schemes occurred, NRCMS and URBMI were combined into a single category, the urban and rural resident’s medical insurance (URRMI), in some provinces.[9] URRMI represents a fourth scheme in China, therefore. It is currently available only in nine of the 31 provinces of China.[10]

Despite this positive progress to ensure medical coverage for all citizens, the Social Insurance Law of the People’s Republic of China states that social medical insurance covers only the cost of medical care for injuries having no legally responsible person/party or injuries for which the legally responsible person/party that caused the injury cannot be reached or does not pay the medical expenses.[11] This national reimbursement requirement allows local governments to develop their own reimbursement details based on the preferences and financial situations in local governments.[12] Consequently, different reimbursement regulations exist across different Chinese provinces and cities.

Our systematic search of both the English and Chinese scientific literatures suggests scholars have written very little concerning the topic of reimbursement for injury-induced medical expenses in China’s social medical insurance schemes. Relevant publications offer only opinions and perspectives rather than presenting research evidence on reimbursement regulations for injury-induced expenses.[13–17] Studies from some other countries do examine empirically the impact of social medical insurance on medical service utilization[18–20] and health outcomes of trauma patients,[18, 21–24] and they suggest expansion of medical insurance accessibility and improved medical insurance benefits in select nations,[25, 26] but they do not specifically address the extent to which reimbursement for injury-induced medical expenses is covered by social medical insurance schemes.

Thus, this study was designed to examine the extent to which national social medical insurance law in China influences the policies of local governments to provide for reimbursement of medical expenses from injuries caused by external responsible persons or parties. To address this question, we conducted a systematic review of legislative documents, assessing whether and how reimbursement for injury-induced medical expenses is addressed in local Chinese social medical insurance schemes.

## Method

This study is based on open-access legal database and does not involve any private information of individuals. The research was approved by the Medical Ethics Committee of Central South University in Changsha, China.

### Document search

#### Databases

In China, national-level legislative documents are classified into three categories: (a) laws issued by the Standing Committee of the National People’s Congress, which have the strongest legislative power;[27] (b) administrative regulations issued by the State Council, which have comparatively strong legislative power;[27] and (c) departmental regulations issued by ministries and commissions under the State Council, which have the weakest legislative power among the three types of national law.[27] In addition, provinces, autonomous regions and municipalities each establish local regulations according to local needs. When there is relevant overlap, local regulations must be consistent with national policy.[28]

For this study, the China National Knowledge Infrastructure (CNKI) (available from: http://law1.cnki.net/law/brief/result.aspx?dbprefix=CLKLP&catalogName=CLKL_CLS) [29] and the Lawyee database (available from: http://www.lawyee.net) [30] were searched to identify national and local legislative documents related to the reimbursement of injury-induced medical expenses in social medical insurance schemes. CNKI and Lawyee are the two most comprehensive academic law databases in China. The CNKI includes more than 910,000 legislative documents and 80,000 legislative updates in the format of laws, administrative regulations, departmental regulations, and local regulations.[29] The Lawyee database consists of all published national and local legislative documents since 1949.[30]

#### Search terms

Our search was limited to four kinds of social medical insurance schemes in China: the UEBMI for employees, NRCMS for rural residents, URBMI for urban residents, and URRMI for urban and rural residents in participating provinces. Disability insurance schemes are not part of the social insurance system according to the People’s Republic of China Social Insurance Law,[11] and therefore were excluded from our document search. Moreover, we did not include social medical insurance for occupational diseases and injuries because they apply only to adults with fixed jobs and most workers in China work on temporary jobs and are not covered by this insurance scheme.[31]

We used a combination of two groups of search terms to conduct our search of documents. Group A consisted of social medical insurance related terms and group B consisted of injury related terms.

Group A covers the full name and abbreviations of the four types of social medical insurance schemes: urban employee basic medical insurance (UEBMI), urban resident basic medical insurance (URBMI), new rural cooperative medical system (NRCMS), and the urban and rural resident medical insurance (URRMI).

Group B includes both general injury and specific injury causes, all based on the classification of external causes of injury recommended by the United States Centers for Disease Control and Prevention.[32] Following the strategy developed by Li et al,[33] we also expanded injury related terms by including the synonyms and near-synonyms of initial terms available in four online Chinese dictionaries (Handian[34], Hanwedadian[35], Iciba[36] and ODict[37]). In addition, we included the term “caused by third person/party” because pilot searches indicated that this term frequently coexisted with general or cause-specific injury in legislative documents. In total, 45 terms were included in Group B.

Combining the terms in Groups A and B, a total of 360 search combinations were used to conduct the document search. Detailed search terms, listed in the original Chinese language, are included in the S1 Appendix.

#### Inclusion criteria

Four criteria were used to determine eligible documents:

1. relevant to the reimbursement for injury-induced medical expenses for any of the four social medical insurance schemes;
2. being national or local legislative documents;
3. being valid current legislation (that is, not a historical document) at the time of searching and screening legislative documents (from October 2016 to February 2017);
4. focusing on the general population in either a national or local jurisdiction.

#### Document searching and screening

The first author (YG) completed the document search, manual screening and information extraction. 20% of initial documents retrieved through the search were selected at random for a second review by the same researcher and the consistency coefficient for the two-round document screening was 99.8% for the CNKI and 99.4% for Lawyee.

### Data extraction and analysis

A group of eight postgraduates studying at the Xiangya School of Public Health, Central South University (including the 1^st^, 2^nd^, 4^th^ and 5^th^ authors) first developed a draft data extract form (including evaluation indicators) and then conducted a pilot evaluation of 157 eligible policy documents. Issues revealed in the pilot data extraction and document evaluation were resolved through email communication and face-to-face group discussion to improve the data extraction form and document evaluation indicators. Next, we used three indicators to assess our outcomes of interest, the inclusion of reimbursement for injury-induced medical expenses in each legislative document, as listed below.

Indicator A: Does the document allow for reimbursement of injury-induced medical expenses? If the document excluded reimbursement for any injury-induced medical expenses, Indicator A was coded as “No”. Otherwise, it was coded as “Yes”.

Indicator B: What type of injury-induced medical expenses are reimbursed by the legislative document? Four options were coded: (1) expenses for injuries having no legally responsible person/party and not caused by self-harm, alcoholism, drug use, or other legal violations; (2) expenses for injuries having a legally responsible person/party but not caused by self-harm, alcoholism, drug use, or other legal violations; (3) expenses for specific injuries, including burns, scalds, animal bites, vaccine-related adverse reactions, and natural disasters not mentioning whether they have a legally responsible person/party; and (4) expenses for injuries are covered, but no detailed requirements are listed.

Indicator C: Does the legislative document include an exception clause allowing for reimbursement of medical expenses from injuries having a legally responsible person/party or caused by self-harm, alcoholism, drug use, or other legal violations? Four options were coded: (1) no exception clause included; (2) an exception clause is present and applies to injuries whose legally responsible person/party cannot be reached; (3) an exception clause is present and applies to situations when the injured person does not receive reimbursement from the legally responsible person/party; (4) an exception clause is present and applies to injuries whose legally responsible person/party cannot be reached or the injured person does not receive reimbursement from the legally responsible person/party.

In addition to collecting data concerning the three indicators, we extracted the following information from all eligible documents: full-text transcription of relevant clauses, name of the document, type of legislative document (law issued by the Standing Committee of the National People’s Congress, regulation issued by the State Council, departmental regulation issued by ministries and commissions under the State Council, or regulation by local government), administrative level (national, provincial, municipal, or county), issue date, and type of social medical insurance scheme or schemes involved (UEBMI, URBMI, NRCMS, and/or URRMI). All potentially related document texts were extracted from each eligible document for formal evaluation.

The evaluation of policy documents was completed by the first author (YG). On rare occasions when the researcher felt she could not make a judgment with confidence, group discussion among nine researchers (including the 1^st^, 4^th^, 5^th^ and 6^th^ authors) was held. This step was required for only 2 of 2878 judgments (0.07%) among the 1038 eligible documents. Note that 1038 documents were evaluated for indicator A, 1012 documents for indicator B and 828 documents for indicator C.

We quantified inclusion of reimbursement for injury-induced medical expenses in included legislative documents using raw numbers and proportions. Subgroup analyses were conducted by type of social medical insurance scheme and by administrative level. Because the data were population-based, inferential statistics were inappropriate and we used descriptive statistics to report all results.

## Results

### Characteristics of included legislative documents

**Fig 1:**
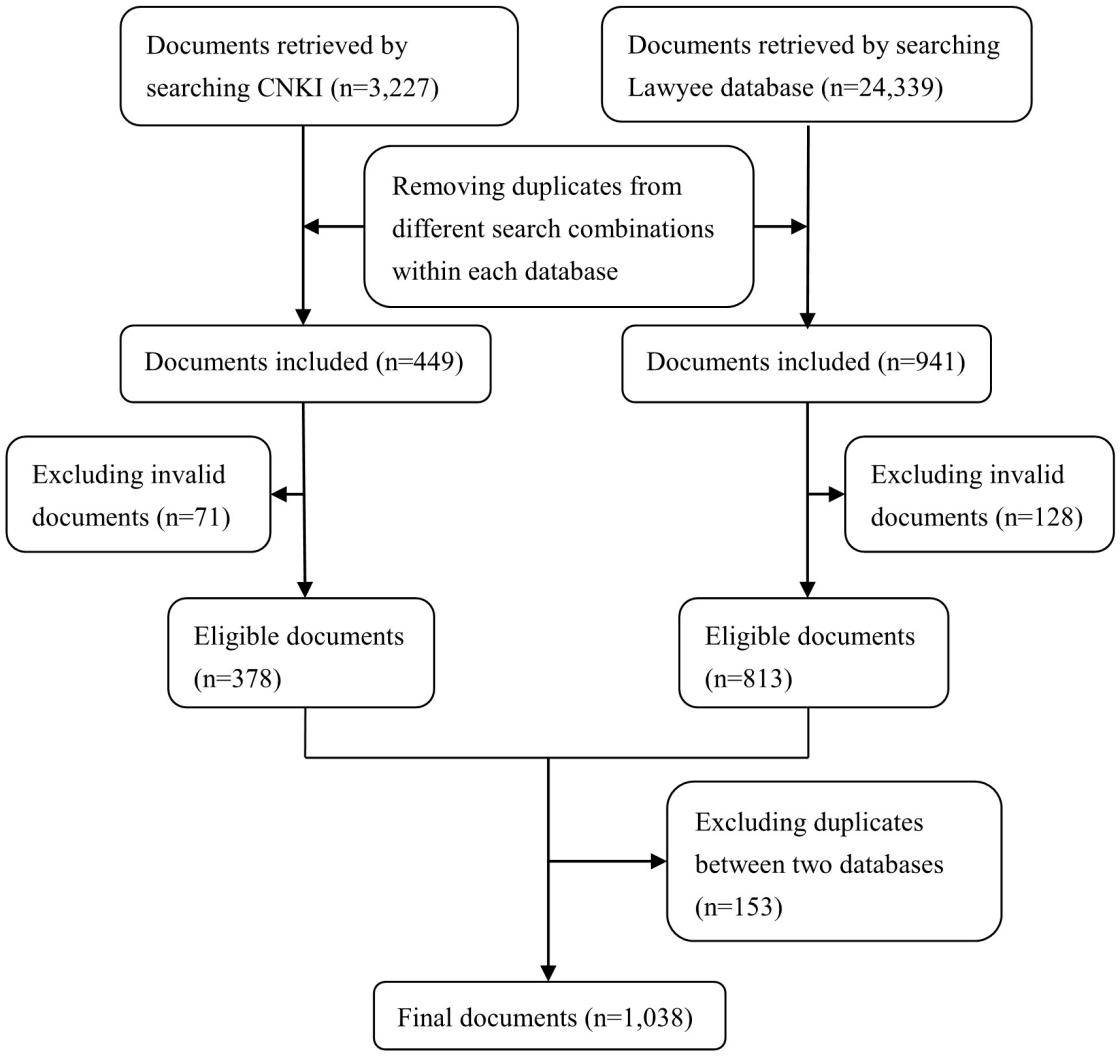
Flow chart of searching legislative documents related to the reimbursement for injury-induced medical expense of four basic social medical insurance schemes of China. CNKI: China National Knowledge Infrastructure. Note: Some invalid documents were searched although “legally valid document” was included as an inclusion criterion.

We retrieved 3,227 and 24,339 legislative documents from the CNKI and Lawyee databases, respectively (Fig 1). In total, 1,038 eligible documents (one national law and 1,037 local regulations) were identified after eliminating duplicate and invalid documents (Table 1). The sole national law addressed reimbursement for injury-induced medical expenses in the UEBMI, URBMI and NRCMS schemes. Respectively, 218, 433, 372 and 80 local regulations addressed reimbursement for UEBMI, URBMI, NRCMS, and URRMI plans (note that these numbers sum to greater than 1037 because some local legislative documents cover reimbursement of two or more types of medical insurance schemes simultaneously).

**Table 1:** Laws/regulations involving reimbursement for injury-induced medical expense in Chinese social medical insurance schemes.

Type of insurance scheme	Administrative level of legislative document			
	National	Provincial	Municipal	County
UEBMI	1	38	145	35
URBMI	1	34	299	100
NRCMS	1	40	104	228
URRMI	0	11	30	39

UEBMI: urban employee basic medical insurance

NRCMS: new rural cooperative medical insurance

URBMI: urban resident basic medical insurance

URRMI: urban and rural resident medical insurance

Note: Some legislative documents involve the reimbursement of two or more kinds of medical insurance schemes simultaneously.

### Document allowing for reimbursement for injury-induced medical expenses

1012 of the 1038 (97%) legislative documents allowed for reimbursement of injury-induced medical expenses (Table 2). Reimbursement patterns were similar across all social medical insurance schemes and administrative levels.

**Table 2:** Legislative documents concerning social medical insurance reimbursement of injury-induced medical expenses, number (percentage).

Administrative level	Type of medical insurance scheme				
	UEBMI	URBMI	NRCMS	URRMI	All schemes
All levels	213(98%)	419(97%)	364(98%)	80(100%)	1012(97%)
National	1(100%)	1(100%)	1(100%)	0(0%)	1(100%)
Provincial	38(100%)	34(100%)	40(100%)	11(100%)	98(100%)
Municipal	141(97%)	289(97%)	100(96%)	30(100%)	531(97%)
County	33(94%)	95(95%)	223(98%)	39(100%)	382(97%)

UEBMI: urban employee basic medical insurance

NRCMS: new rural cooperative medical insurance

URBMI: urban resident basic medical insurance

URRMI: urban and rural resident medical insurance

Note: Some legislative documents involve the reimbursement of two or more kinds of medical insurance schemes simultaneously.

### Detailed reimbursements for injury-induced medical expenses

The majority of the legislative documents (828, or 82%) allowed for reimbursement of medical expenses from injuries having no legally responsible person/party and not caused by self-harm, alcoholism, drug use, or other legal violations (Category A). Most of the remaining documents (162, or 16%) provided no detailed requirements, although they did allow for reimbursement of injury-induced medical expenses (Table 3). Data patterns were similar across all insurance schemes and administrative levels.

**Table 3:** Reimbursement for injury-induced medical expense in Chinese social medical insurance laws/regulations, number (percentage).

Administrative level	Reimbursement	Type of insurance scheme				
		UEBMI	URBMI	NRCMS	URRMI	All schemes
All levels^#^	Category A	190(89%)	321(77%)	299(82%)	66(83%)	828(82%)
	Category B	0	4(1%)	0	1(1%)	5(0.5%)
	Category C	7(3%)	6(1%)	14(4%)	0	17(2%)
	Category D	16(8%)	88(21%)	51(14%)	13(16%)	162(16%)
Provincial	Category A	32(84%)	23(68%)	25(63%)	7(64%)	70(71%)
	Category B	0	0	0	0	0
	Category C	4(11%)	4(12%)	4(10%)	0	5(5%)
	Category D	2(5%)	7(21%)	11(28%)	4(36%)	23(23%)
Municipal	Category A	129(91%)	222(77%)	75(75%)	28(93%)	430(81%)
	Category B	0	3(1%)	0	0	3(1%)
	Category C	2(1%)	0	6(6%)	0	7(1%)
	Category D	10(7%)	63(22%)	19(19%)	2(7%)	91(17%)
County	Category A	28(85%)	75(79%)	198(89%)	31(79%)	327(86%)
	Category B	0	1(1%)	0	1(3%)	2(1%)
	Category C	1(3%)	1(1%)	4(2%)	0	5(1%)
	Category D	4(12%)	18(19%)	21(9%)	7(18%)	48(13%)

Abbreviations of insurance schemes: **UEBMI**, urban employee basic medical insurance; **NRCMS**, new rural cooperative medical insurance; **URBMI**, urban resident basic medical insurance; **URRMI**, urban and rural resident medical insurance.

Type of reimbursement: **Category A**, injuries having no legally responsible person/party and not caused by self-harm, alcoholism, drug use, or other law violations; **Category B**, injuries having legally responsible person/party but not caused by self-harm, alcoholism, drug use, or other law violations; **Category C**, merely specifying injuries from burn, scald, animal bites, vaccine-related adverse reactions, natural disaster but not mention whether to have a legally responsible person/party; **Category D**, injuries with no detailed requirements.

^#^: The results were omitted for the national level because there is only one legislative document involving the four insurance schemes at the same time and their reimbursements for injury-induced medical expenses all belong to category A.

Note: Some legislative documents involve the reimbursement of two or more kinds of medical insurance schemes simultaneously.

### Exception clauses for injuries caused by a legally responsible person/party or by the included risky behaviors

Of the 828 legislative documents not covering injuries with a legally responsible person/party or caused by self-harm, alcoholism, drug use, or other legal violations, 760 (92%) had no exception clause. Twenty-three documents had an exception clause for injuries whose legally responsible person/party could not be reached, 7 had an exception clause in situations where the injured person did not receive reimbursement from the legally responsible person/party, and 38 had an exception clause applying to injuries whose legally responsible person/party could not be reached or when the injured person did not receive reimbursement from the responsible person/party (Table 4). Data patterns were similar across insurance schemes and administrative levels.

**Table 4:** Reimbursement for medical expenses caused by injuries having no responsible person/party and not caused by given reasons in Chinese legislative documents (number/proportion).

Administrative level	Reimbursement	Type of insurance scheme				
		UEBMI	URBMI	NRCMS	URRMI	All schemes
All levels^#^	Subcategory A_1_	170(89%)	304(94%)	265(88%)	55(83%)	760(92%)
	Subcategory A_2_	1(0.5%)	7(2%)	11(4%)	4(6%)	23(3%)
	Subcategory A_3_	2(1%)	1(0.3%)	5(2%)	1(2%)	7(1%)
	Subcategory A_4_	18(9%)	10(3%)	19(6%)	6(9%)	38(5%)
Provincial	Subcategory A_1_	27(84%)	18(78%)	14(56%)	4(57%)	55(79%)
	Subcategory A_2_	0	0	3(12%)	1(14%)	4(6%)
	Subcategory A_3_	0	0	0	0	0
	Subcategory A_4_	5(16%)	5(22%)	8(32%)	2(29%)	11(16%)
Municipal	Subcategory A_1_	114(88%)	217(98%)	63(84%)	24(86%)	400(93%)
	Subcategory A_2_	1(1%)	0	4(5%)	1(4%)	6(1%)
	Subcategory A_3_	2(2%)	1(0.5%)	4(5%)	1(4%)	6(1%)
	Subcategory A_4_	12(9%)	4(2%)	4(5%)	2(7%)	18(4%)
County	Subcategory A_1_	28(100%)	68(91%)	187(94%)	27(87%)	305(93%)
	Subcategory A_2_	0	7(9%)	4(2%)	2(6%)	13(4%)
	Subcategory A_3_	0	0	1(1%)	0	1(0.3%)
	Subcategory A_4_	0	0	6(3%)	2(6%)	8(2%)

Abbreviations of insurance schemes: **UEBMI**, urban employee basic medical insurance; **NRCMS**, new rural cooperative medical insurance; **URBMI**, urban resident basic medical insurance; **URRMI**, urban and rural resident medical insurance.

Four subcategories of reimbursements for medical expenses from injuries having no legally responsible person/party and not caused by given reasons: **Subcategory A**_**1**_: having no exception clause; **Subcategory A**_**2**_: having an exception clause but only applying to situations where the legally responsible person/party for the injury cannot be reached; **Subcategory A**_**3**_: having an exception clause but only applying to situations where the injured person does not receive any reimbursement from the legally responsible person/party; **Subcategory A**_**4**_: having an exception clause applying to situations where the legally responsible person/party for the injury cannot be reached and the injured person does not receive any reimbursement from the responsible person/party.

^#^: The results were omitted for the national level because there is only one legislative document involving the first three insurance schemes and their reimbursements for injury-induced medical expenses all belong to category A_4_.

Note: Some legislative documents involve the reimbursement of two or more kinds of medical insurance schemes simultaneously.

## Discussion

### Main findings

From both moral and logical perspectives, comprehensive social medical insurance programs should cover all health and medical conditions, including those caused by injuries. We found that most Chinese regulations (97%) included reimbursement of injury-induced medical expenses. However, of the 1,012 documents covering injuries, 828 (82%) did not include coverage for injuries having a legally responsible person/party or caused by risky behaviors such as alcohol use, and 16% provided no specific details concerning reimbursement. Further, of the 828 documents not incorporating coverage for injuries having a legally responsible person/party or caused by the included risky behaviors, 92% had no exception clause to account for situations where, for example, the responsible person or party could not be reached or did not pay the expenses.

### Interpretation of findings and their implications

Following previous research,[33] we adopted a systematic set of rigorous scientific strategies to identify relevant legal documents and located 1038 relevant documents. 4% (38 of 1038) of the documents we identified required that social medical insurance provide reimbursement for injury-induced medical expenses when the legally responsible person/party for the injury cannot be reached or does not pay the expenses, including the Social Insurance Law of the People’s Republic of China.[11] However, we identified 760 documents (73% of 1038) that did not allow for such reimbursement and another 26 (2.5%) documents that explicitly excluded reimbursement of any injury-induced medical expenses. Similar results emerged across different types of social medical scheme and administrative levels, demonstrating the widespread and consistent pattern of policies across China. The findings support previous assertions that both the scope (comprehensiveness of services covered) and depth (degree of financial risk protection) of local and regional Chinese social medical insurance are insufficient to maintain public health and inconsistent with published national government mandates.[12]

The exclusion of injuries from social insurance in local legislative documents can have serious impact on injured individuals and their families living in those jurisdictions. In situations where the legally responsible person/party for the injury cannot be reached or refuses to pay the expenses, substantial economic burden could fall to the injured person and his/her family. The situation also can lead to fraud in the medical community, whereby clinical diagnoses are tampered by medical personnel from injuries to other causes to help patients obtain social medical insurance reimbursement.[3,38–40] Beyond ethical responsibilities, such fraud could bias health policy-making by creating inaccurate and misleading health statistics.[41]

Injuries cause approximately 700,000 deaths and 620 million hospitalizations in China each year.[42] From the perspective of justice, we might always strive for medical reimbursement of injury-related expenses for patients and their families from legally responsible person/parties, especially after violent and traumatic injuries such as assaults or road traffic crashes. In many cases, however, the legally responsible parties may not be able to afford the reimbursements and even when they could, it seems unfair to ask injured parties to seek the reimbursement from perpetrators themselves. Such policies restrict injured individuals and their families from obtaining reimbursement from social medical insurance funds in a timely and complete manner, and therefore increase economic burden to them. Because such injuries are more common among low socioeconomic groups in China, and especially among low-income rural residents, the situation may exaggerate health disparities.[43–44] Similar situations appear to exist in other countries worldwide.[45].

Such legal policies also could conflict with the principles of the World Health Organization Global Alliance for Care of the Injured—to ensure essential medical coverage for patients without regards to the ability to pay[46] and achieve universal health coverage for society[2]. Considering that injury control is seriously neglected in many other low- and middle-income countries (LMICs),[47, 48] inadequate reimbursement of injury-induced medical expenses likely exists in countries worldwide. For example, one publication suggests health insurance for injured patients is limited in Vietnam.[25] Some developed countries do cover all injuries in their social medical insurance schemes—such as the United States [49] and United Kingdom[50]–and they could serve as a model for LMICs.

### Limitations

This study was limited by the completeness (representativeness) of national and local legislative documents in the legal document databases that we used. Some local legislative documents may not appear in databases because some local governments retain their legislation as private documents. This is especially the case at the county level, where we detected only 394 eligible documents (there are 1,875 counties and county-level cities in China).[51] This impact is likely to be minimal, however, because county-level legislation typically replicates closely the legislation at provincial and municipal levels.[52] The study was also limited by using just one coder to extract information from the legal documents. The coding was done using objective criteria developed jointly by the research group, and concordance over two rounds of review was very high, but adding a second independent coder would improve research quality in future research.

## Conclusion

We conclude that the majority of Chinese local legislative documents related to social medical insurance do not provide reimbursement for medical expenses from injuries having a legally responsible person/party or caused by illegal behaviors. The results likely derive from the fact that local regulations follow the national social medical law. To realize universal health coverage nationwide, China has two logical options. First, legislators could mandate that all injury-induced medical expenses are reimbursed by social medical insurance at the national level, no matter what the cause of the injury. Further scientific research could support such a policy change by assessing the economic and policy impacts of it, including the acceptability, feasibility and affordability of relevant law and policy changes (e.g. provision situations and constraint requirements; actuarial science to examine benefits and burdens; transfers between urban and rural residents covered by the insurance schemes). An alternative approach would be national availability of supplementary insurance (or subsidy programs) to provide injury insurance, disability insurance, poverty remedy and children’s aid. Such programs, if used widely, could relieve economic burden to injured persons and their families if they allow for reimbursements not offered by primary insurance programs. Of course, the two options also could be integrated to maximally alleviate economic burden to citizens.

## Supporting information

S1 Appendix Search terms in English and in Chinese. (DOCX)
