# Supplementary material for: Reimbursement for injury-induced medical expenses in Chinese social medical insurance schemes: A systematic analysis of legislative documents
Source: PLoS One. 2018 Mar 15;13(3):e0194381. doi: 10.1371/journal.pone.0194381 (PMC5854375; doi:10.1371/journal.pone.0194381)
Supplement: S1 Appendix — (DOCX) [file pone.0194381.s001.docx]

**Search terms in English**

**Group A:** urban employee basic medical insurance, UEBMI, urban resident basic medical insurance, URBMI, new rural cooperative medical system, NRCMS, urban and rural resident medical insurance, URRMI.

**Group B:**injury,harm, damage, infringement, trauma, humiliation, bully, tease, abuse, insult, browbeat, hurt, destruction, kill, slaughter, mutilate, ruin, destroy, affront, drowning, falls, tumble, burn, scald, bite, poisoning, strike, suffocation, road injury, traffic accident, car accident, fight, tussle, fistfight, scuffle, homicide, murder, injury by another, suicide, self-harm, commit suicide, firearm, cut, pierce, undertaken by third party**.**

**Search terms in Chinese**

**Group A:** 城镇职工基本医疗保险，职工医保，城镇居民基本医疗保险，居民医保，新型农村合作医疗，新农合,城乡居民基本医疗保险，城乡居民医保。

**Group B:**伤害，危害，损害，侵害，外伤，欺辱，欺凌，虐待，欺负，欺侮，迫害，损伤，摧残，残害，杀害，毁伤，糟蹋，损坏，侮辱，溺水，跌倒，摔倒，烧伤，烫伤，咬伤，中毒，撞击，窒息，道路交通伤害，交通事故，车祸，打架，斗殴，相打，打斗，他杀，谋杀，他伤，自杀，自戕，自伤，枪伤，刺伤，割伤，第三人负担。
